# Photorefractive Keratectomy with Adjunctive Mitomycin C for Residual Error after Laser-Assisted *In Situ* Keratomileusis Using the Pulzar 213 nm Solid-State Laser: Early Results

**DOI:** 10.1155/2013/815840

**Published:** 2013-09-28

**Authors:** Maya Fe Ng-Darjuan, Raymond P. Evangelista, Archimedes Lee D. Agahan

**Affiliations:** ^1^Refractive Surgery Service, Manila Vision Correction Center, Ermita, 1004 Manila, Philippines; ^2^Department of Ophthalmology and Visual Sciences, Sentro Oftalmologico Jose Rizal, Philippine General Hospital, University of the Philippines Manila, Taft Avenue, 1000 Manila, Philippines

## Abstract

*Purpose*. To evaluate the accuracy, efficacy, stability, and safety of photorefractive keratectomy (PRK) enhancement using the Pulzar 213 nm solid-state laser (SSL) with adjunctive Mitomycin C in eyes previously treated with laser assisted *in situ* keratomileusis (LASIK) with residual error of refraction. *Methods*. This is a prospective noncomparative case series of 16 eyes of 12 patients who underwent PRK for residual refractive error after primary LASIK. Mitomycin C 0.02% was used after the PRK to prevent haze formation. Outcomes measured were pre- and postoperative manifest refraction spherical equivalent (MRSE), uncorrected (UDVA) and best-corrected distance visual acuity (CDVA), and slit lamp evidence of corneal complications. *Results*. The mean UDVA improved from 20/70 preoperatively to 20/30 postoperatively. The average gain in lines for the UDVA was 2.38. After six months of followup, the postoperative MRSE within 0.50 D in 56% (9) of eyes and 94% (15) eyes were within 1.0 diopters of the intended correction. No eyes developed haze all throughout the study. *Conclusion*. PRK enhancement with adjunctive use of Mitomycin C for the correction of residual error of refraction after LASIK using the Pulzar 213 nm solid-state laser is an accurate, effective, and safe procedure.

## 1. Introduction

Laser eye surgery has been accepted worldwide as a procedure to modify the shape of the cornea and correct myopia, hyperopia, astigmatism, and presbyopia. However, the cornea is not a plastic material that if shaped a certain way would retain that shape forever. The cornea is a living tissue wherein its biomechanical and wound healing properties can restrict the predictability and stability of refractive surgery [1]. These factors contribute to the discrepancies between intended and achieved visual outcomes after laser-assisted *in situ* keratomileusis (LASIK), surface ablation, and other keratorefractive procedures leading to residual errors. To correct the remaining refractive error, a second refractive laser surgery can be done. In this study, we chose to do photorefractive keratectomy (PRK) with adjunctive Mitomycin C using the 213 nm solid-state laser for the correction of residual error after LASIK. Photorefractive keratectomy (PRK) with adjunctive Mitomycin C has been shown to be safe and effective with the use of the 193 nm excimer lasers. These lasers have been widely used in the past two decades and up to the present [2–4]. With the recent development and introduction of the 213 nm solid-state laser, use of this machine for refractive surgery has been increasing in various parts of the world. The two lasers have different properties, and while studies have shown that the clinical results between the two are comparable when performing primary LASIK and PRK [5–7], these differences may produce varying results when applied onto a previously created LASIK flap. The 213 nm laser has greater transmissibility through water and balanced salt solution and is closer to the peak absorption of corneal collagen [8, 9]. Theoretically, the 213 nm solid-state laser allows more selective energy absorption by corneal collagen and less energy absorption by the surrounding water. The corneal surface is notably dried during ablation using the 193 nm excimer whereas it becomes moist during ablation using the 213 nm solid-state laser. It could not be established yet whether 213 nm laser is more cytotoxic and/or mutagenic compared to 193 nm laser because of limited data. However, several studies have proven that corneal ablation by both 213 nm and 193 nm wavelengths produces minimal DNA damage and free radical formation [10, 11]. The 213 nm has also been determined to deliver less energy on the ablation surface, therefore producing less thermal effect than the 193 nm laser [10].

To our knowledge, this is the first study on the safety and efficacy of performing PRK enhancement procedure on post-LASIK eyes using the Pulzar Z1 213 nm solid-state laser.

## 2. Materials and Method

This is a prospective interventional case series done in an outpatient refractive surgery center in Manila, Philippines. Sixteen (16) post-LASIK eyes with residual error of refraction requiring enhancement were subjected to PRK using the Pulzar Z1 SSL. Mean retreatment period was 7 ± 8.52 months (range: 1–36 months) after the primary LASIK were performed. All eyes have previously undergone LASIK using the Pulzar Z1 SSL. The epithelium was subjected to 20% ethyl alcohol using a well for 50 seconds. The epithelium was removed to the periphery beyond the edges of the LASIK flap. Topography guided laser ablation was performed, and 0.02% Mitomycin C was applied on to the corneal bed for 50 seconds using a soaked sponge. The cornea was washed generously with BSS. Clear bandage contact lens was placed. Standard PRK medications consisted of Moxifloxacin every 4 hours and Prednisolone acetate every 8 hours for two weeks. The bandage contact lens was removed after seven days. The manifest refraction spherical equivalent (MRSE), uncorrected visual acuity (UDVA), and best-corrected distance visual acuity (CDVA) were taken at 1 week, 1 month, 3 months, and 6 months. The eyes were monitored for the development of haze. Only patients and eyes completing the 6-month followup were included in the study.

## 3. Results

### 3.1. Demographic

 A total of 16 eyes of 12 patients underwent PRK due to residual error of refraction.  Table 1 shows the baseline data of all patients included in the study. All patients underwent bilateral primary LASIK (using the Pulzar 213 nm SSL) then retreated with PRK for enhancement. The interval between primary LASIK to PRK enhancement procedure was between 4 weeks and 3 years. The aim of the enhancement procedure is to remove all or significantly reduce the residual error of refraction from the previous LASIK. Shown in Table 1 are the preoperative manifest refraction spherical equivalent (MRSE) of −1.41 ± 1.43 (range: −4.93 to 0.82) and preoperative astigmatism of −1.28 ± 1.00 (range: −3.87 to 0.12) we treated in this study. No intra- or postoperative complications occurred during primary LASIK or PRK. The patients were monitored up to 6 months after enhancement procedure.

### 3.2. Visual Acuity

**Figure 1:**
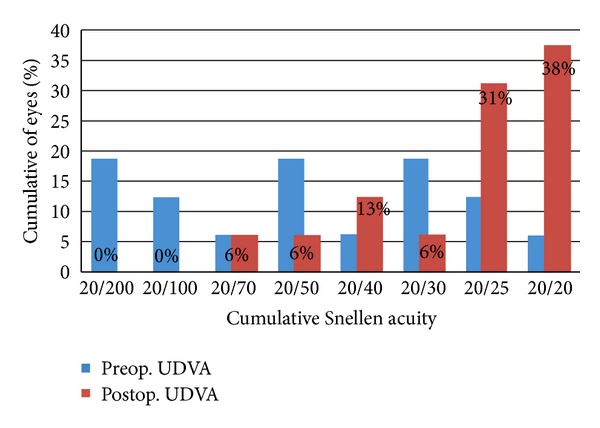
Cumulative uncorrected distance visual acuity pre-Prk and post-Prk.

 The mean visual acuity of the patients improved from a preoperative UDVA of logMAR 0.46 ± 0.34 (≈20/70) to postoperative UDVA logMAR 0.14 ± 0.16 (≈20/30). At the last followup, 11/16 (69%) of the eyes had a postoperative UDVA of 20/25 or better after enhancement procedure while 14/16 (88%) of the eyes had postoperative UDVA of 20/40 or better. All eyes (100%) had a vision of 20/70 or better postoperatively (Figure 1).

As shown in Table 2, fourteen out of sixteen eyes (87%) showed improvement in the UDVA, gaining between 1 and 6 lines after PRK enhancement with 7/15 (44%) gaining 3 to 4 lines, 6/16 (38%) gaining 1 to 2 lines, and 1/16 (6%) gaining 5 to 6 lines. The average gain in lines for the UDVA is 2.38. Two out of the sixteen eyes did not show improvement in UDVA. None of the eyes had worsened UDVA after the procedure.

### 3.3. Accuracy of Correction

**Figure 2:**
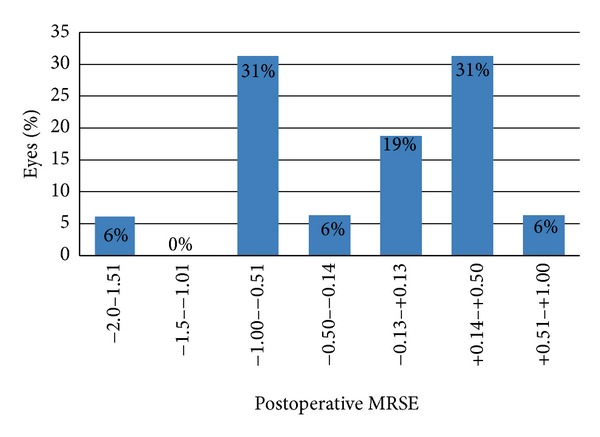
Manifest refractive spherical equivalent (MRSE) 6 months after PRK.

**Figure 3:**
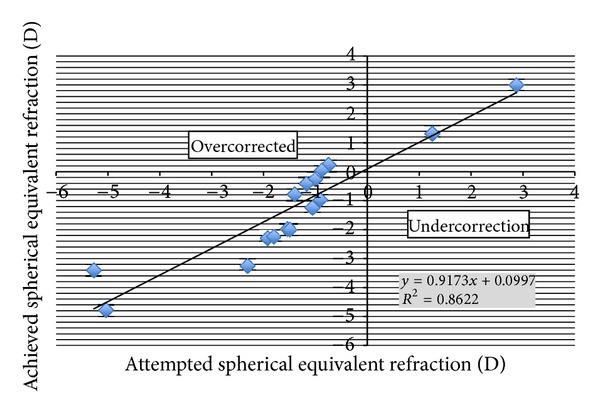
Correlation graph between target and achieved SE. Correlation coefficient = 0.86.

 After six months of followup, the postoperative MRSE were within 0.50 D in 56% (9) of eyes and 94% (15) eyes were within 1.0 D. Only one eye (6%) was noted to have a residual of more than 1.0 D difference from that of target (Figure 2). The mean attempted enhancement correction is −1.40 ± 1.93 and the mean achieved enhancement correction is −1.18 ± 1.91. The linear regression analysis shows a very slight tendency towards undercorrection (slope = 0.9173; intercept = 0.0997) (Figure 3). Coefficient of determination revealed a strong correlation between the attempted and the achieved correction (*R* ^2^ = 0.86).

### 3.4. Stability

**Figure 4:**
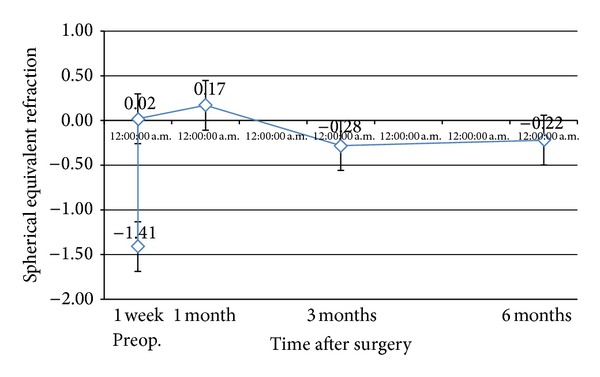
Mean MRSE pre-PRK and throughout the 6-month followup.

The postoperative refraction of the eyes treated remained stable all throughout the 6-month follow-up period.  Figure 4 shows that the mean MRSE went close to emmetropia on the first week after enhancement then regressed slightly by 0.2 D on the last followup. 

### 3.5. Safety

**Figure 5:**
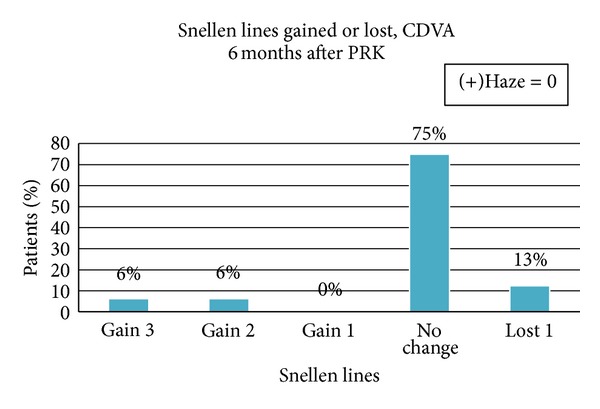
Distribution of eyes as to the Snellen lines gained or lost after PRK enhancement.

Figure 5 shows fourteen out of sixteen (87%) eyes treated either showed no change or gained lines in their CDVA. Two eyes lost 1 line (one from 20/25 to 20/30 and the other from 20/20 to 20/25) in their CDVA. No eyes lost 2 or more lines in their CDVA. None of the eyes developed haze of any degree during the six-month follow-up period.

**Figure 6:**
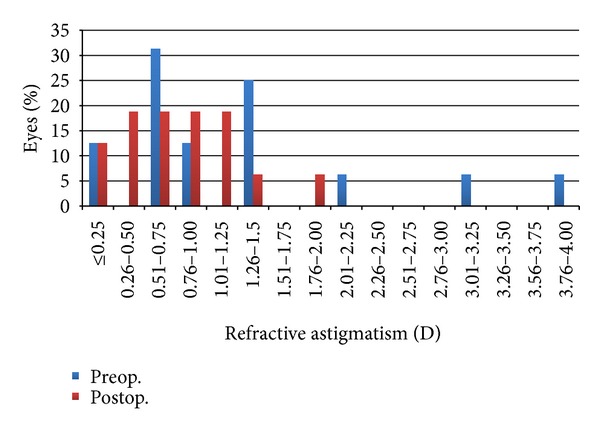
Cumulative refractive astigmatism before and after enhancement.

Figure 6 shows the varied astigmatism of patients before and after the enhancement procedure. The mean preoperative cylinder was −1.28 ± 1.00 D, and the mean cylinder six months after enhancement was −0.84 ± 0.48 D. The mean change in cylinder power was −0.44 ± 0.73 D (range: −1.87 to 0.00). Thirteen percent (2) of eyes had induced astigmatism of −0.62 ± 0.18 D higher than baseline. 69% (11) of the eyes had decreased astigmatism of −0.76 ± 0.63 D at the end of the follow-up period. There was no change in astigmatism in 19% (3) of eyes.

Prior to enhancement procedure, 25% (4) of eyes had against the rule astigmatism in which half remained against the rule and the other half rotated to an oblique axis with mean change of 21° rotation after enhancement. Thirteen percent (2) of eyes had with the rule astigmatism preoperatively where one became against the rule and the other became oblique in axis with mean change in rotation of 95°. 62% (10) of eyes had oblique astigmatism at the start of the study where in 80% (8) remained oblique, 10% (1) became against the rule and another 10% (1) became with the rule, at the end of the follow-up period.

## 4. Discussion

LASIK has become the procedure of choice globally for laser correction of errors of refraction. However, enhancement procedures are sometimes done to improve unexpected results due to several factors like residual errors, flap complications, and patient satisfaction. In this study, we focused on treating the residual errors of the patients who underwent primary LASIK. We utilized the 213 nm solid-state laser PRK enhancement. Photorefractive keratectomy enhancement decreases the risk of epithelial ingrowth, and it dramatically reduces corneal ectasia.

Adjunctive use of Mitomycin C with PRK after primary LASIK has been shown to be effective and safe using 193 nm excimer lasers. With the development and growing use of the 213 nm solid-state laser, published studies [12–14] have suggested that despite the known differences in their ablation characteristics, clinical results in efficacy and safety have been comparable. In a study by Sanders et al., the authors showed that cellular responses after irradiation with 213 nm compared with 193 nm wavelengths are consistent with good clinical outcomes [11]. However, laser ablation of a LASIK flap presents unique problems because of delayed epithelial healing and remodeling as well as stromal changes that may have resulted from previous denervation [15].

Although the safety and efficacy of using the Pulzar Z1 213 nm SSL for this procedure have not been evaluated prior to this study, theoretically this machine may be superior than an excimer laser system in terms of elimination of toxic gas use. In addition to that, the Pulzar Z1 laser system is able to create smooth ablation surface because of its small spot size, fast tracking system, high pulse to pulse stability, and standardized Gaussian intensity beam distribution [16]. Greater transmissibility through water and BSS of 213 nm solid-state lasers makes treatment results less susceptible to the effects of corneal hydration and environmental humidity which could affect the final outcome from the usual excimer laser system. Hence, ablation with a 213 nm wavelength may result in better wound healing, leading to a more reliable correction of refractive errors. In our study, we have evaluated the efficacy, accuracy, safety, and stability of photorefractive keratectomy using the Pulzar Z1 SSL to correct residual errors after LASIK. 

In this study, we used Mitomycin C 0.02% as an adjunctive treatment to prevent corneal haze. Prevention of corneal haze with the of use Mitomycin C 0.02% is already a widely accepted procedure [17, 18]. Previous studies by Benito-Llopis and Teus and Muller et al. showed similar results using the same Mitomycin C concentration used in this study. These studies revealed no delay in re-epithelialization and no eyes experienced haze [19, 20]. Taneri et al. were even able to remove epithelial ingrowth from a previous buttonholed laser *in situ* keratomileusis and prevent haze formation by doing epithelial removal with phototherapeutic keratectomy, application of Mitomycin C for 1 minute followed by myopic PRK [18]. 

In this study, 16 eyes out of 12 subjects were included. All patients previously underwent primary LASIK which resulted in residual error with MRSE ranging from −4.93 to 0.82. The attempted MRSE correction of −1.40 ± 1.93 resulted in a slight undercorrection (mean −0.22 ± 0.93) ending up with an achieved MRSE of −1.19 ± 1.91. Linear regression analysis proved a strong correlation between the attempted and the achieved correction (*R* ^2^ = 0.86). This result shows that PRK with 213 nm solid-state laser is useful and effective in treating residual errors after primary LASIK.

The uncorrected distance visual acuity (UDVA) of the eyes treated showed that 69% had UDVA of 20/25 or better, 88% had UDVA of 20/40 or better, and 100% had UDVA of 20/70 or better. The majority (87%) of eyes showed significant improvement in vision with an average of 2.38 lines gained after PRK six months after the enhancement procedure. No eye lost a line for UDVA following the procedure. These results are comparable to those of previous studies which utilized excimer laser [17, 21–23]. Shaikh followed up 15 eyes that underwent PRK after LASIK for 6 months. He reported that after 6 months of followup, 87% of eyes had UDVA of ≥20/40, 53% had ≥20/25, and 40% had ≥20/20. In our study, the UDVA (6 months after PRK) was 20/40 or better in 88% of eyes and 69% had 20/25 or better. Authors such as Neira, Srinivasan, and Beerthuizen reported that PRK enhancement after LASIK with the 193 nm excimer laser is effective and safe. This study suggests that using a 213 nm solid-state laser for PRK enhancement is as effective as the 193 nm excimer laser system in providing improvement in UDVA for eyes with residual errors after LASIK. 

The greater number of eyes treated either gained (12%) or remained the same (75%) in their CDVA after treatment. While two eyes lost one line in CDVA, the UDVA improved in both treated eyes. The eye that lost one line from 20/25 to 20/30 in CDVA gained 3 lines in UDVA from 20/100 to 20/40. The other eye that lost 1 line in CDVA from 20/20 to 20/25 also gained 2 lines in UDVA from 20/70 to 20/40. Loss of one line in two eyes could be due to corneal high aberration induction leading to reduction of image quality. Degree of axis rotations between preoperative and postoperative astigmatism is not significantly related to the amount of achieved correction and visual acuity of the patients.

None of the eyes developed any degree of haze during the 6-month follow-up period possibly as a result of adjunctive use of Mitomycin C. The postoperative refraction of the treated eyes remained stable throughout the 6-month follow-up period. These findings proved that 213 nm solid-state laser PRK is safe to use with predictable results.

It is recommended that results of a longer follow-up period should be sought for better understanding of this procedure. Furthermore, future studies should take into account other factors such as induced corneal wavefront aberrations, corneal topographic changes, patient satisfaction, and visual outcomes. 

## 5. Conclusion

PRK enhancement with adjunctive use of Mitomycin C for the correction of residual error of refraction after LASIK using the Pulzar 213 nm solid-state laser is an effective, accurate, predictable, and safe enhancement procedure.

## Figures and Tables

**Table 1:** Demographics and baseline data.

Parameter	Values
Patients : Eyes (*n*)	12 : 16
Male : Female	4 : 8
Age (y)	
Mean ± SD	42 ± 15.87
Range	26 to 80
Pre-operative UDVA (log⁡MAR )	
Mean	0.46 ± 0.33
Range	0.00 to +1.00
Pre-operative CDVA (log⁡MAR)	0.04 ± 0.10
Mean	0.04 ± 0.10
Range	0.00 to +0.30
Pre-operative MRSE (D)	
Mean ± SD	−1.41 ± 1.43
Range	−4.93 to 0.82
Preoperative astigmatism (D)	
Mean ± SD	−1.28 ± 1.0
Range	−3.87 to −0.12
Attempted correction (D)	
Mean ± SD	−1.40 ± + 1.93
Range	−5.28 to +2.87
Indication for PRK	Residual error of refraction
Mean interval of LASIK to PRK	7 ± 8.52 months (Range: 1 to 36 months)
Length of followup	6 months

CDVA: corrected distance visual.

MRSE: manifest refraction spherical equivalent.

UDVA: uncorrected distance visual acuity.

**Table 2:** Lines improved, UDVA.

Lines improved	Number	%
0	2	13
1 to 2	6	38
3 to 4	7	44
5 to 6	1	6

Total	16	100

## References

[1] Dupps WJ, Wilson SE (2006). Biomechanics and wound healing in the cornea. *Experimental Eye Research*.

[2] Alió JL, Piñero DP, Plaza Puche AB (2008). Corneal wavefront-guided photorefractive keratectomy in patients with irregular corneas after corneal refractive surgery. *Journal of Cataract and Refractive Surgery*.

[3] Chalita MR, Roth AS, Krueger RR (2004). Wavefront-guided surface ablation with prophylactic use of mitomycin C after a buttonhole laser in situ keratomileusis flap. *Journal of Refractive Surgery*.

[4] Liu A, Manche EE (2010). Visually significant haze after retreatment with photorefractive keratectomy with mitomycin-C following laser in situ keratomileusis. *Journal of Cataract and Refractive Surgery*.

[5] Tsiklis NS, Kymionis GD, Kounis GA, Naoumidi II, Pallikaris IG (2008). Photorefractive keratectomy using solid state laser 213 nm and excimer laser 193 nm: a randomized, contralateral, comparative, experimental study. *Investigative Ophthalmology and Visual Science*.

[6] Dair GT, Pelouch WS, van Saarloos PP, Lloyd DJ, Paz Linares SM, Reinholz F (1999). Investigation of corneal ablation efficiency using ultraviolet 213-nm solid state laser pulses. *Investigative Ophthalmology and Visual Science*.

[7] Ren Q, Simon G, Legeais J-M (1994). Ultraviolet solid-state laser (213-nm) photorefractive keratectomy: in vivo study. *Ophthalmology*.

[8] Dair GT, Ashman RA, Eikelboom RH, Reinholz F, van Saarloos PP (2001). Absorption of 193- and 213-nm laser wavelengths in sodium chloride solution and balanced salt solution. *Archives of Ophthalmology*.

[9] Tsiklis NS, Kymionis GD, Kounis GA, Naoumidi II, Pallikaris IG (2008). Photorefractive keratectomy using solid state laser 213 nm and excimer laser 193 nm: a randomized, contralateral, comparative, experimental study. *Investigative Ophthalmology and Visual Science*.

[10] van Saarloos P, Jain M, Pujara T Carcinogenetic and mutagenic action of 193-nm, 213-nm and 266-nm laser radiation.

[11] Sanders T, Pujara T, Camelo S (2009). A comparison of corneal cellular responses after 213-nm compared with 193-nm laser photorefractive keratectomy in rabbits. *Cornea*.

[12] Tsiklis NS, Kymionis GD, Kounis GA (2007). One-year results of photorefractive keratectomy and laser in situ keratomileusis for myopia using a 213 nm wavelength solid-state laser. *Journal of Cataract and Refractive Surgery*.

[13] Shah S, Sheppard AL, Castle J (2012). Refractive outcomes of laser-assisted subepithelial keratectomy for myopia, hyperopia, and astigmatism using a 213 nm wavelength solid-state laser. *Journal of Cataract and Refractive Surgery*.

[14] Quito CFG, Agahan ALD, Evangelista RP (2013). Long-term follow-up of laser in-situ keratomileusis for hyperopia using a 213 nm wavelength solid-state laser. *ISRN Ophthalmology*.

[15] Javier J, Charukamnoetkanok P, Azar DT (2004). Basic aspect of corneal wound healing. *Wave Front Customized Visual Correction: Quest for Super Vision II*.

[16] Tsiklis NS, Kymionis GD, Kounis GA (2007). One-year results of photorefractive keratectomy and laser in situ keratomileusis for myopia using a 213 nm wavelength solid-state laser. *Journal of Cataract and Refractive Surgery*.

[17] Srinivasan S, Drake A, Herzig S (2008). Photorefractive keratectomy with 0.02% mitomycin C for treatment of residual refractive errors after LASIK. *Journal of Refractive Surgery*.

[18] Taneri S, Koch JM, Melki SA, Azar DT (2005). Mitomycin-C assisted photorefractive keratectomy in the treatment of buttonholed laser in situ keratomileusis flaps associated with epithelial ingrowth. *Journal of Cataract and Refractive Surgery*.

[19] de Benito-Llopis L, Teus MA (2010). Efficacy of surface ablation retreatments using mitomycin C. *American Journal of Ophthalmology*.

[20] Muller LT, Candal EM, Epstein RJ, Dennis RF, Majmudar PA (2005). Transepithelial phototherapeutic keratectomy/photorefractive keratectomy with adjunctive mitomycin-C for complicated LASIK flaps. *Journal of Cataract and Refractive Surgery*.

[21] Beerthuizen JJG, Siebelt E (2007). Surface ablation after laser in situ keratomileusis: retreatment on the flap. *Journal of Cataract and Refractive Surgery*.

[22] Neira-Zalentein W, Moilanen JAO, Tuisku IS, Holopainen JM, Tervo TMT (2008). Photorefractive keratectomy retreatment after LASIK. *Journal of Refractive Surgery*.

[23] Shaikh NM, Wee CE, Kaufman SC (2005). The safety and efficacy of photorefractive keratectomy after laser in situ keratomileusis. *Journal of Refractive Surgery*.

